# Robotic‐assisted bone cuts guided by intraoperative load sensors for ligament balancing in restricted kinematic alignment total knee arthroplasty: A retrospective study with 2‐year follow‐up

**DOI:** 10.1002/jeo2.70780

**Published:** 2026-05-22

**Authors:** Julien Bardou‐Jacquet, Sonja Cabarkapa, Jérôme Murgier

**Affiliations:** ^1^ Clinique Tivoli‐Ducos, Institut de chirurgie robotique Euratlantique Bordeaux France; ^2^ Saint‐Vincent's Hospital Melbourne Victoria Australia; ^3^ Clinique Aguiléra, service de chirurgie orthopédique, ramsay santé Biarritz France

**Keywords:** ligament balancing, load sensors, robotic arm, soft‐tissue, total knee arthroplasty

## Abstract

**Purpose:**

Achieving optimal ligament balance during total knee arthroplasty (TKA) improves patient satisfaction. While kinematic alignment enhances ligament balance compared to mechanical alignment, soft tissue releases are sometimes necessary. However, soft tissue releases lack consistency and reproducibility, contributing to suboptimal outcomes despite the use of intraoperative load sensors. Recent studies suggest tibial recuts as an alternative method for ligament balance. This study investigates the feasibility and efficacy of robotic‐assisted bone recuts for achieving ligament balance, with a secondary aim of determining a force threshold correlating with negative clinical outcomes.

**Methods:**

A retrospective study of 109 patients undergoing TKA between February 2020 and February 2021 was conducted. All surgeries used Stryker Triathlon implants with no soft tissue releases. Bone recuts, in 0.5‐mm increments, were performed at the tibia or femur based on intraoperative load sensor feedback. Clinical outcomes were assessed using the Forgotten Joint Score 12 (FJS‐12) and the Knee Injury and Osteoarthritis Outcome Score 12 at 2 years. Eight patients were lost to follow‐up at 2 years.

**Results:**

At the end of the procedure, 92% of knees were balanced at 10° of flexion, and 85% were balanced at 90° of flexion. After tibial recuts, the medial femorotibial force decreased by an average of 33.6 lbf at 10° of flexion and 30.1 lbf at 90° of flexion. At 2 years, the mean FJS‐12 score was 78.94, with 65% of patients achieving a score above 77.1. No significant correlation was found between intraoperative ligament balance and clinical outcomes.

**Conclusion:**

Robotic‐assisted ligament balancing using bone recuts is a feasible and reproducible technique to achieve intraoperative balance without soft tissue releases. However, improved ligament balance did not result in superior clinical outcomes at 2 years.

**Level of Evidence:**

Level III, retrospective comparative study.

**Abbreviations**
CR: cruciate retaining
FJS‐12: Forgotten Joint Score 12
KOOS‐12: Knee Injury and Osteoarthritis Outcome Score 12
LBF: pound‐forces
PASS: patient acceptable symptom state
TKA: total knee arthroplasty

## INTRODUCTION

Ligament balancing improves patient satisfaction during total knee arthroplasty (TKA) [8]. Implant positioning using kinematic alignment enhances ligament balance compared to mechanical alignment [13]. However, soft tissue releases are sometimes necessary to achieve a balanced knee [12]. Soft tissue releases do not provide consistent or reproducible results [10], which may explain the lack of improvement in patient satisfaction despite the use of intraoperative force sensors [3, 4, 5, 6]. A recent study demonstrated the potential for achieving ligament balance through tibial bone recuts of 2 mm instead of peri‐articular ligament releases, under the guidance of an intraoperative force sensor [23]. However, balancing the knee in both flexion and extension solely through tibial bone recuts without soft tissue releases remains challenging [12].

The primary objective of this study was to assess the feasibility and efficacy of ligament balance using femoral (in the coronal or horizontal plane) and/or tibial bone recuts (in the coronal plane) performed with robotic assistance under intraoperative force sensor control. The secondary objective was to determine a threshold force value measured by the sensor, above which functional outcomes would be negatively impacted at 2 years post‐operatively.

The main hypothesis was that Robotic‐assisted ligament balancing using bone recuts is a feasible and reproducible technique to achieve intraoperative balance without soft tissue releases.

## MATERIALS AND METHODS

### Study design and patients

This was a single‐centre study investigating data collected prospectively and analysed retrospectively of a consecutive, prospective series of patients undergoing TKA between February 2020 and February 2021. All surgeries were performed by the same experienced surgeon skilled in robotic‐assisted ligament balancing using intraoperative force sensors. The study received ethical approval from the Research Ethics Committee of the University Hospital of Bordeaux under the registration number 2230561v0 on 10 July 2023. Inclusion criteria were patients undergoing primary TKA for osteoarthritis (Kellgren–Lawrence stage 3/4), excluding those with a history of fracture, infection or previous partial/TKA.

The study included 109 patients (53 females, 55 males), with a mean age of 70 years (range, 45–89). The mean body mass index (BMI) was 29.2 kg/m^2^ (range, 19–45). All patients were non‐smokers at the time of surgery. There were 36 patients with a history of knee surgery on the operated side (30 meniscectomies, 6 ACL reconstructions, 4 high tibial osteotomies and 1 patellar ligament reconstruction). There were 30 patients excluded due to the unavailability of the force sensor at the time of surgery, and 8 patients were lost to follow‐up at 2 years.

### Surgical technique and intraoperative measurements

All patients received identical implants, Stryker Triathlon (Stryker), cementless, with posterior cruciate ligament retention and cruciate‐retaining (CR) polyethylene. All patellae were resurfaced, and no soft tissue releases were performed. The surgical approach was medial parapatellar. Preoperative planning was based on restricted kinematic alignment (±6° varus/valgus), with intraoperative adjustments to implant positioning, as described by Shatrov et al. [20] and Clark et al. [2], to achieve an 18‐mm flexion and extension space both medially and laterally, regardless of deformity. After trial implants were placed, a force sensor (Verasense, Stryker) was used. The arthrotomy was temporarily closed using towel clips [26], and measurements were taken as per the protocol described by Meneghini et al. [14].

Ligament balancing was achieved exclusively through tibial bone recuts in the coronal plane and/or femoral recuts in the coronal or axial planes. Bone recuts were performed in 0.5‐mm increments, with force sensor feedback after each recut as described in a previous study [1]. The goal was to achieve ligament balance according to Gustke et al.'s criteria [9], with forces between 5 and 45 pound‐forces (lbf) and a mediolateral difference of less than 15 lbf. Final implants were impacted without further force measurement prior to closure.

### Clinical follow‐up

All patients were re‐evaluated by an independent operator 2 years post‐operatively. Patient satisfaction was assessed using the Forgotten Joint Score 12 (FJS‐12) [22] and the Knee Injury and Osteoarthritis Outcome Score 12 (KOOS‐12) [3].

### Data analyses

Statistical analyses were primarily descriptive for patient demographics. Categorical variables were presented as counts and percentages. Continuous variables were presented as means and standard deviations (SDs). Pearson's chi‐square analyses were used to determine statistical significance for categorical variables, and the McNemar chi‐square test for matched samples. For continuous variables, the Shapiro–Wilk tests were performed to assess for normality between the two variables. If negative, the random behaviour of the residuals was checked graphically, and a quantile‐by‐quantile plot of the re‐normalised residuals was drawn close to the first bisector to graphically validate the normality hypothesis, along with a plot of the histogram of the residuals to check the normality of the random error term epsilon. The continuous variables, which were shown to demonstrate normality, were assessed using Welch's *t* tests, whereas those that did not meet this criterion were compared using Mann–Whitney *U* tests. Different FJS‐12 thresholds were considered to determine the patient acceptable symptom state (PASS; >33.3) and the ‘forgotten joint status’ (>77.1) (FJS PASS) [22]. An analysis of variance (ANOVA) was used to compare the means of the clinical scores (FJS‐12 and KOOS‐12) with whether or not the knees were well balanced at the end of the procedure. All statistical analyses were performed using the software known as RStudio (R Foundation for Computational Statistics). And a *p* value < 0.05 was considered statistically significant [5].

## RESULTS

### Ligament balancing and surgery

**Figure 1:**
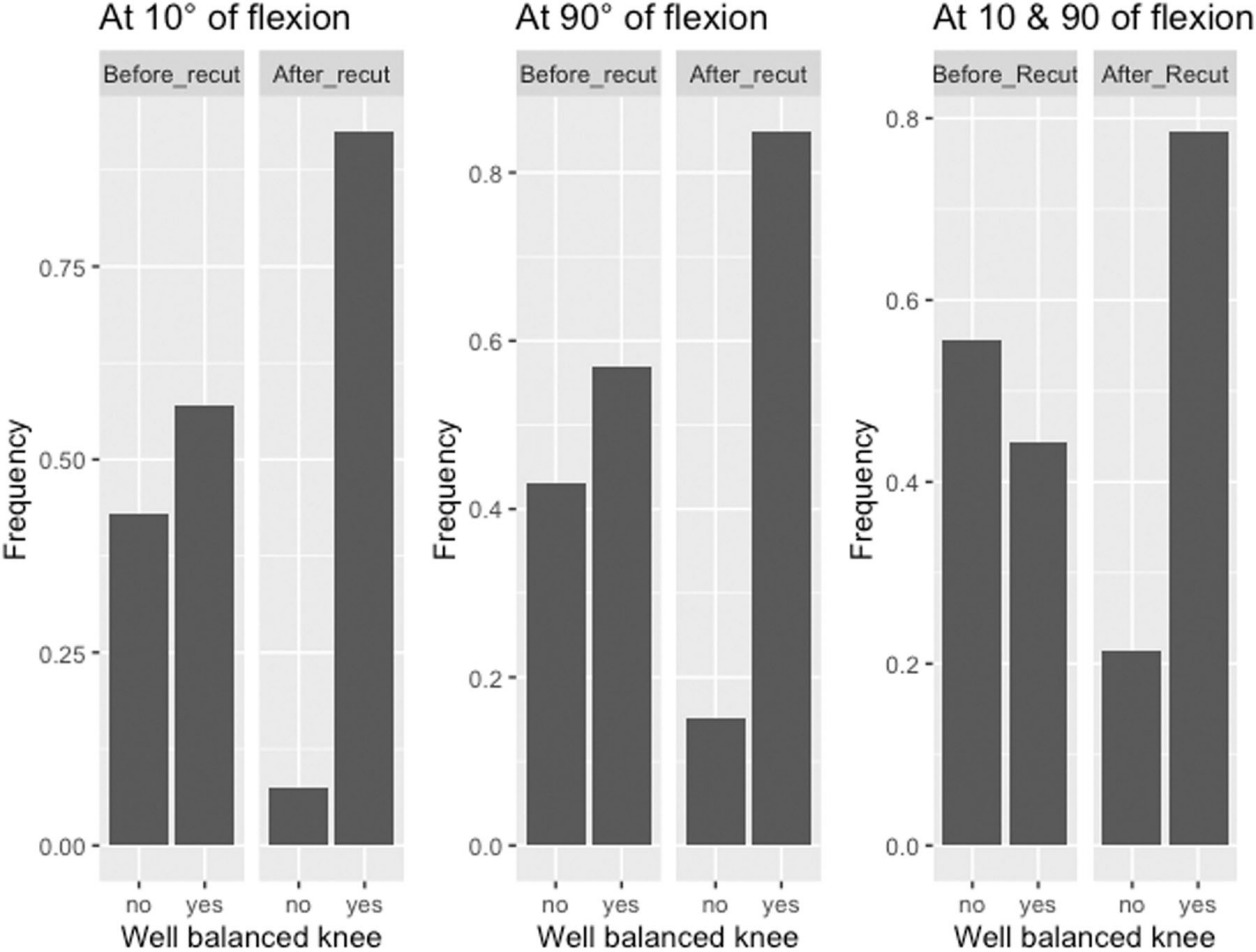
Frequency of well‐balanced knees before and after bone recut at different degrees of flexion.

At the end of the procedure, 92% of knees were balanced at 10° of flexion (compared to 57% pre‐recut), 85% were balanced at 90° of flexion (compared to 57% pre‐recut), and 79% of knees were balanced at both 10° and 90° of flexion (compared to 44% pre‐recut). In all cases, the difference in balance before and after bone recuts was statistically significant (*p* < 0.001) (Figure 1).

**Figure 2:**
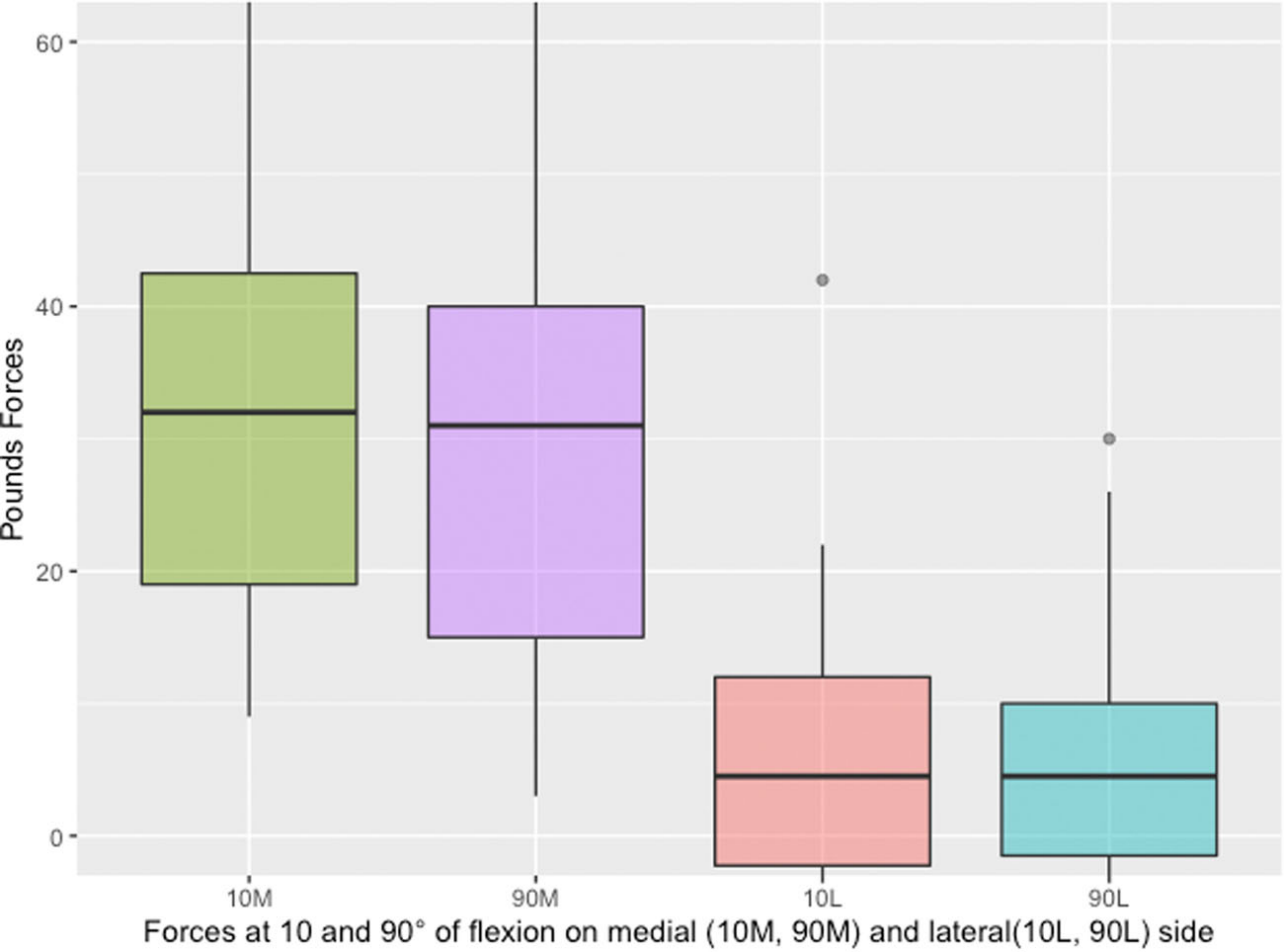
Distribution of the difference in forces measured between the femur and the tibia before and after a 0,5° varus tibial frontal bone recut as a function of the medial or lateral side and the degree of flexion.

On average, a tibial recut of 0.5° in the coronal plane reduced medial femorotibial force by 33.6 lbf (SD = 19.5) at 10° of flexion and by 30.1 lbf (SD = 18.1) at 90° of flexion, while lateral force changed by only 5.4 lbf (SD = 13.4) at 10° of flexion and 5.7 lbf (SD = 11.9) at 90° of flexion (Figure 2). Other values are shown in Table 1.

**Table 1:** Force difference measured by the sensor between the femur and tibia, depending on the type of bone recut.

Type of recut	Degree of knee flexion	Side of measure	Number of cases	Mean (lbf)	Standard deviation (lbf)
Tibial 0.5 varus recut	10°	Medial	19	33.6	19.5
		Lateral	19	5.4	13.4
	90°	Medial	19	30.1	18.1
		Lateral	19	5.7	11.9
Tibial 0.5 valgus recut	10°	Medial	9	8.7	12.6
		Lateral	9	33.2	17.3
	90°	Medial	9	10.1	12.2
		Lateral	9	33.1	18.0
Femoral 0.5 varus recut	10°	Medial	3	22.0	21.3
		Lateral	3	9.0	4.6
	90°	Medial	3	1.7	8.1
		Lateral	3	7.3	10.7
Femoral 0.5 valgus recut	10°	Medial	4	−7.3	4.8
		Lateral	4	13.8	9.1
	90°	Medial	4	0.0	6.6
		Lateral	4	6.3	4.8
Femoral 0.5 external rotation	10°	Medial	11	7.6	5.7
		Lateral	11	2.1	5.2
	90°	Medial	11	23.6	12.3
		Lateral	11	−0.6	8.6

*Note*: Lbf = pound forces.

**Figure 3:**
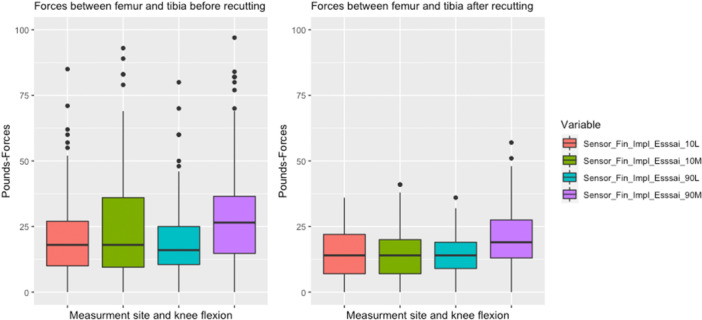
Distribution of forces measured between the femur and the tibia on the lateral and medial sides at 10° and 90° of flexion. Sensor_Fin_Impl_Esssai_10L = Load at 10° of flexion on the lateral side. Sensor_Fin_Impl_Esssai_10M = Load at 10° of flexion on the medial side. Sensor_Fin_Impl_Esssai_90L = Load at 90° of flexion on the lateral side. Sensor_Fin_Impl_Esssai_90M = Load at 90° of flexion on the medial side.

The percentage of knees classified as ‘too tight’ (force exceeding 45 lbf according to Gustke et al. criteria [9]) was 34.2% before any recut and 3.8% after recut at the end of the procedure. The distribution of forces before and after balancing recut is shown in Figure 3.

### Ligament balancing and outcomes

There were 71 patients available for follow‐up at 2 years. The mean FJS‐12 score was 78.94 (SD = 25.6; range, 0–100). KOOS‐12 Pain averaged 87.28 (SD = 18.8; range, 25–100), KOOS‐12 Function averaged 87.07 (SD = 21.4; range, 19–100), and KOOS‐12 Quality of Life averaged 84.9 (SD = 23.9; range, 0–100).

Regarding FJS PASS, 46 patients (65%) had a score >77.1, 20 patients (28%) had scores between 77.1 and 33.3, and 5 patients (7%) had scores <33.3.

There was no statistically significant correlation between intraoperative knee balance and clinical outcomes. No threshold force value was identified that negatively impacted clinical results (Table 2).

**Table 2:** Statistical relationships between clinical results and intraoperative forces.

Variables compared	Type of variable	Type of test	*p* < 0.05	*p*	Pearson correlation coefficient
FJS12_PASS_77 vs. Balanced knee	Qualitative vs. Qualitative	Chi‐squared test	No	0.63	–
FJS12_PASS_33 vs. Balanced knee		Fisher's exact test	No	0.58	–
FJS12 vs. Balanced knee	Quantitative vs. Qualitative	ANOVA	No	0.2	–
FJS12 vs. Balanced knee at 10° of flexion		ANOVA	No	0.24	–
FJS12 vs. Balanced knee at 90° of flexion		ANOVA	No	0.4	–
FJS12 vs. Force on medial side at 10° of flexion	Quantitative vs. Quantitative	Pearson linear correlation test	No	0.31	−0.12
FJS12 vs. Force on lateral side at 10° of flexion		Pearson linear correlation test	No	0.16	−0.16
FJS12 vs. Force on medial side at 90° of flexion		Pearson linear correlation test	No	0.47	0.09
FJS12 vs. Force on lateral side at 90° of flexion		Pearson linear correlation test	No	0.14	−0.18
FJS12 vs. difference in medio‐lateral force at 10° of flexion	Quantitative vs. Quantitative	Pearson linear correlation test	No	0.67	0.05
FJS12 vs. difference in medio‐lateral force at 90° of flexion		Pearson linear correlation test	No	0.57	0.07

*Note*: Definitions: FJS12_PASS_77: Forgotten Joint Score 12 > 77. FJS12_PASS_33: Forgotten Joint Score 12 < 33.

Abbreviations: FJS12, Forgotten Joint Score‐12; PASS, patient acceptable symptom state.

## DISCUSSION

The most important finding was that the combined use of force sensors and robotic‐assisted technology significantly improved ligament balance in TKA without the need for peri‐articular ligament releases. This was achieved reliably and reproducibly and represents the first study to demonstrate this possibility using restricted kinematic alignment planning.

However, this study did not establish a statistical correlation between intraoperative knee balance and clinical outcomes at 2 years. No force threshold beyond which clinical outcomes worsened was found. The goal of intraoperative ligament balancing during TKA is to create rectangular, equal spaces in both flexion and extension after tensioning the peripheral ligaments. Excessive ligament tension should be avoided to prevent a tight joint that can cause pain (overstuffing) [25]. Intraoperative functional alignment, through manual tensioning of peri‐articular ligaments, creates these rectangular spaces [4]. This technique improves balance and reduces the need for ligament releases [17, 18, 19]. However, even with this technique and the same evaluation criteria, 50% of knees in this study were deemed unbalanced. One possible explanation is that manual ligament tensioning, even by an experienced surgeon, is neither reliable nor reproducible without feedback from an intraoperative force sensor [14]. This sensor only provides information after bone cuts have been made [15]. Thus, there is a need to find a way to balance ligament tension after bone cuts, either through soft tissue releases or additional bone recuts. Peri‐articular soft tissue releases are difficult to control and may result in over‐release, even under the guidance of a force sensor [10]. Tibial bone recuts are a feasible method for balancing, but they do not address all situations (such as flexion balance) and may still require ligament releases [23]. This study showed that it is possible to balance peri‐articular ligament tension through bone recuts as small as 0.5 mm at the femur and/or tibia, without performing any soft tissue releases. Each 0.5‐mm recut resulted in a homolateral reduction in femorotibial force of approximately 30 lbf. Minimal recuts resulted in substantial changes in compartmental forces, highlighting the precision and sensitivity of this technique. The precision of this technique doubled the rate of balanced knees and avoided knees that were too tight or too lax.

Ligament balancing is one of the strategies explored to improve patient satisfaction following TKA [8]. However, the use of intraoperative force sensors has yielded contradictory results depending on the surgical techniques employed. If the sensor is used only for informational purposes, it does not seem to improve clinical outcomes [17, 21]. In cases where ligament balancing involves soft tissue releases, these releases can cause pain [24] or result in knees that are too lax [10].

This study demonstrated that ligament balance can be achieved during surgery without the need for peri‐articular soft tissue releases, thus avoiding the disadvantages associated with ligament releases. However, no statistical relationship was found between intraoperative knee balance and clinical outcomes at 2 years of follow‐up. Additionally, no link was found between a tight medial femorotibial compartment and better clinical outcomes, as reported by Geller et al. [6]. Differences in implant design may explain these contradictory findings. In this study, the femoral implants used did not feature changes in the femoral curvature radius [21], which theoretically maintains constant space between the femur and tibia throughout the arc of flexion. In Geller et al.'s study, the implants had a decreasing radius of curvature with knee flexion, resulting in increased space in flexion [11]. This difference may explain the need for tighter medial compartments in order to reproduce the natural kinematics of a medial‐pivot knee [21]. In contrast to findings by Meneghini et al. [14], no threshold force value was identified that resulted in poorer clinical outcomes in this study.

## LIMITATIONS

This study has several limitations. It was a retrospective study, which makes it vulnerable to selection bias and confounding factors. Although all surgeries were performed by the same surgeon, which adds consistency, this also limits the generalisability of the results. The study's relatively small sample size may have contributed to the lack of statistically significant findings. A post hoc analysis revealed that for a difference of more than 15 points on the FJS‐12 with a SD of 25, each group would need to include 51 patients to achieve 80% power at an alpha level of 0.05. However, only five patients had an FJS‐12 score below 33.3, which was insufficient to draw robust conclusions. Moreover, no threshold force value was found beyond which clinical outcomes deteriorated. The group of patients with forces above 66 lbf was too small to allow for meaningful comparisons. Nevertheless, eliminating knees that are too tight, which may lead to arthrofibrosis [7], and those that are too lax, which may result in pain [18], could help improve patient satisfaction with this technique. Another criticism is that intraoperative sensor data do not reflect weight‐bearing load [15]. The forces measured during surgery reflect ligament tension [16], but since the implants are non‐deformable compared to living structures, there is little variation in ligament tension during weight‐bearing activities. While this has not yet been studied, it can be assumed that the forces measured intraoperatively under non‐weight‐bearing conditions provide a viable reflection of ligament tension during everyday activities. Also, no adjustment for multiple comparisons was applied. Given the exploratory nature of the study, results should be interpreted with caution. Finally, there was no control group to compare our results with other techniques.

## CONCLUSION

This study demonstrated the feasibility and efficacy of robotic‐assisted ligament balancing using bone recuts at the femur and/or tibia, without soft tissue releases, in achieving balanced knees in TKA. However, no statistical link was found between intraoperative balance and clinical outcomes at 2 years. Further research with larger sample sizes is necessary to determine whether more precise ligament balancing could improve long‐term outcomes and patient satisfaction.

## AUTHOR CONTRIBUTIONS

Julien Bardou‐Jacquet designed and practised the surgery, collected the data and took part in article writing and critical re‐editing. Sonja Cabarkapa did the supervision and proof read the paper. Jérôme Murgier took part in article writing and critical re‐editing.

## CONFLICT OF INTEREST STATEMENT

Julien Bardou‐Jacquet is a consultant for Stryker. The other authors declare no conflicts of interest.

## ETHICS STATEMENT

The study was conducted in accordance with the Declaration of Helsinki. The Research Ethics Committee of Bordeaux issued a favourable opinion for the publication of this research (ref CER‐BDX‐2022‐27). Informed consent was obtained by surgeons for all patients.

## Data Availability

The data sets generated and/or analysed during the current study are not publicly available due to ethical restrictions related to participant confidentiality, but are available from the corresponding author on reasonable request.
